# Is Social Distancing Policy Effective in Controlling COVID-19? An Interrupted Time Series Analysis

**DOI:** 10.22037/aaem.v9i1.1201

**Published:** 2021-05-25

**Authors:** Mehdi Yaseri, Rahim Soleimani-Jelodar, Zohreh Rostami, Saeed Shahsavari, Mostafa Hosseini

**Affiliations:** 1 Department of Epidemiology and Biostatistics, School of Public Health, Tehran University of Medical Sciences, Tehran, Iran. E-mail: m.yaseri@gmail.com; 2 Department of Virology, School of Public Health, Tehran University of Medical Sciences, Tehran, Iran. E-mail: rahimsoleimani1361@yahoo.com; 3 Department of Accounting, Qazvin Branch, Islamic Azad University, Qazvin, Iran. E-mail: rostamizohreh@yahoo.com

**Keywords:** Physical distancing, incidence, COVID-19, interrupted time series analysis, Iran

## Abstract

**Introduction:**

The social distancing plan is one of the ways that was implemented for management of COVID-19 pandemic. This study aimed to evaluate the effect of the social distancing on reducing the daily new cases and deaths from COVID-19.

**Methods:**

In this cross-sectional study, the data of daily new cases and daily deaths were collected from 15/02/2020 to 19/04/2020. Changes in the level and trend of daily new cases and daily deaths before and after the implementation of social distancing plan were evaluated using interrupted time series (ITS) analysis in STATA software.

**Results:**

The post-intervention trend had a decrease of 102 new cases per day and 7 new deaths per day compared to the pre-intervention trend (p < 0.001). Moreover, in the post-intervention period, the daily new cases had a decrease of 58 new cases per day and 2 new deaths per day (p < 0.001).

**Conclusion:**

It Could be concluded that social distancing plan directly affects the new daily cases and new daily deaths.

## 1. Introduction

**Figure 1:**
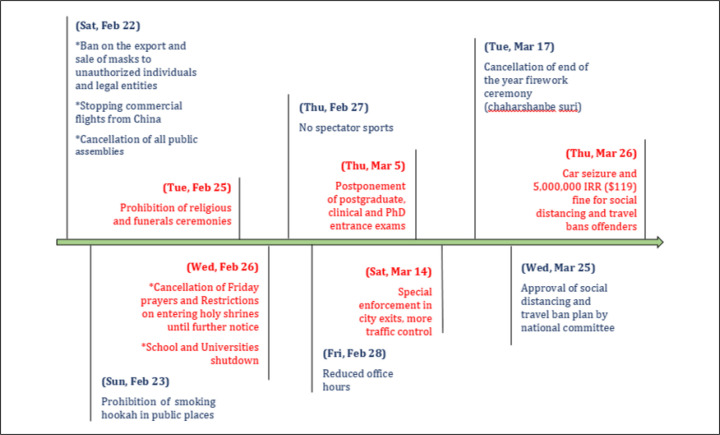
Chronological timeline of Iran’s measures after COVID-19 outbreak

An outbreak of pneumonia with unknown etiology occurred in late December 2019, in Wuhan city, China (1). On Jan 12, 2020, the causative agent of Wuhan pneumonia was temporarily called Novel Coronavirus-2019 (2019-nCoV) by the World Health Organization (WHO) and the resulting disease was named COVID-19 (2). On 19 February 2020, Iran reported its first confirmed cases of SARS-CoV-2 infection in Qom. Raoofi et al. reported the timeline of Iran’s governance measures (figure 1) after the official declaration of the COVID-19 epidemic in Iran (3). Social distancing plan is one of Iran’s governance measures, which was implemented on 25 March, 2020. Detailed data on the effectiveness of non-pharmaceutical interventions (NPIs) are still limited; therefore, conducting public health studies for studying their effect on COVID-19 incidence is necessary. Agarwal et al. studied COVID-19 pandemic and its societal impact and found that race, culture, level of education, and socio-economic status have a major impact on disease outbreak (4). Recently, Pan et al. reported in JAMA, the epidemiological effects of NPI implementation throughout the COVID-19 epidemic in Wuhan, and found that use of multidimensional NPIs had contributed to control of the COVID-19 outbreak in Wuhan (5). Since, COVID-19 outbreak is a serious threat to public health and healthcare personnel universally, it seems essential to control the burden of disease through adoption and implementation of proper policies including social distancing, isolation of COVID-19 patients, and quarantine operation (6). The primary aim of social distancing plan in Iran was to decrease the daily new cases and deaths due to COVID-19 outbreak. It was expected to prevent the collapse of the healthcare system as happened in Italy, Spain, India, and United Kingdom (7-9). Based on the above- mentioned points, this study aimed to evaluate the effect of the social distancing on reducing the number of daily new cases and deaths due to COVID-19.

## 2. Methods

***2.1. Design and study population***

This is a descriptive-analytical study, which has been performed longitudinally. We performed a quasi-experimental, interrupted time series (ITS), as a strong quasi-experimental design to detect immediate and longitudinal changes in COVID-19 daily new cases and deaths in Iran, before and after the social distancing plan. ITS was applied to evaluate the effects of health interventions, ITS is a powerful and increasingly popular design for evaluating public health interventions (10-13). However, it is difficult to fully account for all the biases that might have impacted the COVID-19 daily new cases and deaths in Iran in ways not related to social distancing policy. Autocorrelation condition effect should also be considered when performing this plan: autocorrelation is usually present in the time series data and it is defined as the correlation between the response variable at time t and the response variable at times t-1 and t-2 (13). 

***2.2. Data Sources and data setting***

The daily new cases and deaths were obtained from governmental websites and European center for disease prevention and control between March 21 and April 20, 2020 (14). In the present study, the response variable was daily new cases and daily deaths, the explanatory variable was time (setting day 1 as the first COVID-19 diagnosed case), the intervention variable was social distancing plan in Iran, which was carried out on 25 May 2019 (setting 0 as the period without social distancing between February 15 and March 28, 2020 and 1 as the period with social distancing between March 29 and April 19, 2020) and interaction between time and social distancing plan.

***2.3. Statistical methods***

We applied ITS analysis to assess both the immediate-level changes as well as changes in the trend of the daily new cases and deaths before and after the intervention. Moreover, we created the scatter plot of new daily cases and new deaths over time to visually inspect our data and examine whether there was a social distancing plan effect. Autocorrelation was evaluated through visual detection of the plot of the residuals vs. time, using Durbin-Watson test (15). P-value less than 0.05 was considered to indicate statistical significance. STATA version 14 was used for our data analysis. The segmented regression models are fitted in the form of the least-squares regression line. It is assumed that there is a linear relationship between the time and the response variable inside each segment (11). 

## 3. Results

The impact of the social distancing plan on daily new cases in Iran was reduction in both new daily cases and new death rates.

***3.1. The Impact of social distancing plan on daily new cases***

**Figure 2:**
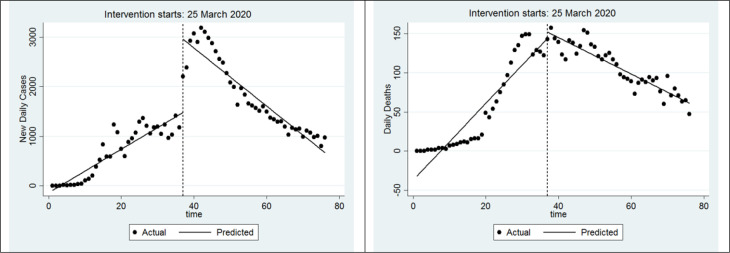
Coronavirus daily new cases (left) and deaths (right) before and after the implementation of social distancing plan in Iran

The results of segmented regression for the response variable of daily new cases demonstrated that daily changes in the number of new cases were significant compared to before implicating social distancing plan (Coef. = 43.6, 95%CI: 36.7 - 50.6; p <0.001). Furthermore, on the day after the intervention, an increase of 1478 cases was observed in the number of new cases (Coef.= 1478.8, 95%CI: 1132.9 – 1824.8; P<0.001). The post-intervention new cases trend had displayed a decrease of 102 cases per day compared to the pre-intervention trend (Coef.= -102.4, 95%CI: -116.4 – -88.3; P<0.001). Moreover, after the intervention, daily new cases had a decrease of 58 cases per day (Coef.= -58.7, 95%CI: -70.4 – -46.9; P<0.001). Figure 2 displays the distribution of COVID-19 daily new cases in Iran before and after the implementation of social distancing plan from February 15, 2020 to May 3, 2020.

***3.2. The*** ***Impact of social distancing plan on daily new deaths***

The results of segmented regression for the Impact of social distancing plan on daily new deaths in Iran demonstrated that daily changes in the new deaths were statistically significant compared to before the social distancing plan implementation (Coef.= 4.8, 95%CI: 3.9 – 5.7; p<0.001). On the next day after the intervention, an increase of about 8 cases was observed in new deaths, which was not significant (Coef.= 7.9, 95%CI: -13.9 – 29.9; p=0.471). The post-intervention new deaths trend showed a decrease of 7 cases per day (compared to the pre-intervention trend). Decrease in the rate of new deaths after intervention was significant (Coef.= -7.2, 95%CI: -8.1 – -6.2; p<0.001). Moreover, after the intervention, the daily new deaths had a decrease of 2 cases per day, which was statistically significant (Coef.= -2.3, 95%CI: -2.6 – -1.9; p<0.001). Figure 2 displays the distribution of COVID-19 daily deaths in Iran before and after the social distancing plan implementation.

## 4. Discussion

This study was conducted to answer this question: Was social distancing policy effective in controlling COVID-19 in Iran? Results of an interrupted time series analysis proved that social distancing plan was significantly effective in reducing the total number of the new daily cases and the new deaths in Iran. As depicted in the results section, daily change in the number of new cases and new deaths was increasing before the implementation of social distancing plan. Furthermore, the trend of daily new cases after the intervention displayed a decrease of 102 cases per day. Also, the trend of new death cases showed a decrease of 7 case per day, on average (compared to the pre-intervention trend). The reasons for this achievement can be the implementation of social distancing as well as the increase in the knowledge levels of individuals. Therefore, social distancing plan seems to be effective in reducing the spread of COVID-19. The primary goal of social distancing plan is to reduce physical contact between individuals and prevent person-to-person spread of the disease (3). Use of non-pharmaceutical public health interventions such as isolation, quarantine, social distancing, and community containment to control infectious disease outbreaks is eﬀective enough to slow down the spread of the virus (2, 16). In a study by Priyadarsini et al., social distancing was introduced as a key factor that could suppress the viral spread directly or indirectly (17). As demonstrated in the results section, social distancing plan implementation had a significant effect on the number of new daily cases and new death cases. There are several studies published in a short time span, indicating the importance of social distancing measures. The same finding has been reported in others’ researches, a study conducted by Medeiros de Figueiredo et al. (18), which had evaluated the impact of lockdown on COVID-19 incidence and mortality in Chinese provinces (Hubei and Guangdong) using ITS model, showed that social distancing measures were effective in reducing the incidence and mortality rates. In another ITS model study by Siedner et al. (19), the mean daily COVID-19 growth rate decreased after implementation of the first statewide social distancing measures. It is essential to mention that although social distancing seems to have been effective in controlling COVID-19 in Iran, there are socio-economic challenges associated with this mechanism. Since this plan closes some jobs and reduces working hours, it can lead to significant productivity losses in the economy in the long run. According to the global epidemic scenario, gross domestic product (GDP) can be reduced by 2.5 percent and 1.8 percent in developing countries and in industrialized countries, Respectively (20). On the other hand, the closing of schools and the cessation of basic education can cause damage in terms of improving the level of basic knowledge in the long run. Thus, many public policymakers may face the challenge of cost-effectiveness of social distancing policy. Finding cost-effective mechanisms in controlling the disease was a key step before the coronavirus vaccine was discovered. 

As evident in the obtained results, the trend of new daily cases and new daily deaths was decreasing after the implantation of social distancing plan. Therefore, it can be concluded that the social distancing plan directly affected the daily new cases and daily new deaths, resulting in a decline in the rates of COVID-19 cases. Furthermore, formulating appropriate policies, such as social distancing, and implementing them seem to be necessary to slow the spread of infection. Thus, social distancing can be used as an effective strategy to save lives and slow the spread of COVID-19. 

## 5. Limitations

It should be noted that this study has some limitations. First, researchers had to use daily new cases data of the disease due to a lack of data on the incidence and prevalence. Second, due to the lack of a comparable control group, the effect of intervening variables and other unobservable variables on the trend of new cases was not controlled. Furthermore, the interrupted time series design defines a simple pre-post comparison that cannot provide information about the various mechanisms leading to the changes.

## 6. Conclusion:

The results of our analysis showed that the trend of daily new cases of COVID-19 and the resulting deaths was decreasing since the implementation of social distancing plan in Iran. In other words, social distancing plan has been effective in controlling this disease. However, such a plan can lead to economic and social challenges. It is suggested that further studies examine the effectiveness of the social spacing scheme by controlling the intervening variables. Studies on the cost-effectiveness of this policy can also provide valuable insight regarding its usefulness.

## 7. Declarations

### 7.1. Acknowledgments

The authors would like to thank all researchers and health professionals that are confronting the COVID-19 pandemic.

### 7.2. Author contribution

MY, SS and MH conceptualized the research idea, collected data, performed the analysis and wrote the first draft of the manuscript. RSJ and ZR contributed in the data acquisition, analysis, interpretation. All authors read and approved the final manuscript.

### 7.3. Conflict of Interest

Authors declared no conflict of interest.

### 7.4. Funding and support

This research received no specific grant from any funding agency in the public, commercial, or not-for-profit sectors.
